# Integrated information theory reveals the potential role of the posterior parietal cortex in sustaining conditioning responses in classical conditioning tasks

**DOI:** 10.3389/fnins.2025.1512724

**Published:** 2025-01-29

**Authors:** Tien Cuong Phi, Shin Ishii, Masashi Kondo, Masanori Matsuzaki, Ken Nakae

**Affiliations:** ^1^Department of Systems Science, Graduate School of Informatics, Kyoto University, Kyoto, Japan; ^2^Neural Information Analysis Laboratories, Advanced Telecommunications Research Institute International, Kyoto, Japan; ^3^International Research Center for Neurointelligence, The University of Tokyo, Tokyo, Japan; ^4^Department of Physiology, Graduate School of Medicine, The University of Tokyo, Tokyo, Japan; ^5^Department of Biological Sciences, Graduate School of Science, The University of Tokyo, Tokyo, Japan; ^6^Brain Functional Dynamics Collaboration Laboratory, RIKEN Center for Brain Science, Saitama, Japan; ^7^Exploratory Research Center on Life and Living Systems, National Institutes of Natural Sciences, Okazaki, Japan

**Keywords:** posterior parietal cortex, classical conditioning, integrated information theory, task performance, network analysis, core complex analysis

## Abstract

Classical conditioning is a fundamental associative learning process in which repeated pairings of a conditioned stimulus (CS) with an unconditioned stimulus (US) lead to the CS eliciting a conditioned response (CR). Previous research has identified key neural regions involved in processing reward-predicting cues and mediating licking behavior. However, the mechanisms that sustain high conditioned response rates across repeated sessions remain elusive, particularly regarding how the reward expectation is represented on a session-by-session basis. While early learning phases in classical conditioning have been extensively studied, the neural mechanisms that support consistent performance over time remain unclear. In this study, we sought to understand how cortical regions, particularly the posterior parietal cortex (PPC), contribute to maintaining high CR rates across sessions. Using the core complex framework derived from Integrated Information Theory (IIT), we explored the dynamics of neural networks during sessions of high CR performance. Our findings suggest that while traditional functional connectivity (FC) methods struggled to capture the complexity of sustained behavioral engagement, the core complex framework revealed key regions, notably the PPC, that were significantly correlated with enhanced CR sessions. This work suggests the potential role of the PPC in supporting reward expectations and maintaining consistent behavioral responses. By applying the core complex framework to investigate neural substrates of sustained behavior, we provide novel insights into the interaction of cortical networks during classical conditioning, offering promising directions for future research in associative learning and behavior.

## 1 Introduction

Classical conditioning is a core associative learning process in which a conditioned stimulus (CS) is repeatedly paired with an unconditioned stimulus (US), eventually leading to the CS eliciting a conditioned response (CR) in the absence of the US. Previous studies have identified key neural regions involved in processing reward prediction cues, encoding reward prediction errors, tracking reward history, outcome history, and mediating licking behavior (Bloem et al., 2017; Kondo and Matsuzaki, 2021; Oyama et al., 2015; Shin et al., 2018; Yoshizawa et al., 2018). However, the mechanisms that sustain high conditioned response rates across repeated sessions remain poorly understood, especially how reward expectation is represented on a session-by-session basis.

While much is known about the initial learning phases in classical conditioning, less is understood about how multiple cortical regions interact to maintain consistent performance across sessions. Classical conditioning is not simply a reflex; it engages a network of brain regions that integrate sensory input and motor output (Banno et al., 2020; Kondo and Matsuzaki, 2021). In particular, it is not clear how reward expectation is encoded in the cortical areas that link the primary sensory and motor cortices. It is also unknown whether these cortical activities reflect recognition of CS, motivation to perform the task, or both, especially over repeated sessions. Furthermore, previous research has primarily focused on neural circuits involved in immediate reward processing (Bloem et al., 2017; Kondo and Matsuzaki, 2021; Oyama et al., 2015; Shin et al., 2018; Yoshizawa et al., 2018), but sustained licking behavior engagement across sessions likely involves more complex interactions between cortical regions. Understanding how these systems interact over repeated sessions is essential for understanding how conditioned behaviors are sustained.

Traditional approaches mainly focus on pairwise or linear relationships, which may fail to capture the full complexity of neural interactions (Fornito et al., 2016; Lahaye et al., 2003; Li, 2022). Integrated Information Theory (IIT) (Albantakis et al., 2023; Oizumi et al., 2014; Tononi, 2004; Tononi et al., 2016) offers a framework for understanding how cognitive processes such as motivation and recognition emerge from integrated brain networks or even complex cognitive process such as consciousness (Boly et al., 2017; Casarotto et al., 2016; Rosanova et al., 2012; Sarasso et al., 2014; Tsuchiya et al., 2015). We applied the “core complex” framework (Kitazono et al., 2018; Kitazono et al., 2020), which identifies highly interconnected regions involved in high-level cognitive functions. There exist several definitions of “core” in functional connectivity studies, which use either graphical or informational approaches, such as maximal cliques (Ercsey-Ravasz et al., 2013), k-cores (Chatterjee and Sinha, 2007; Hagmann et al., 2008; Harriger et al., 2012; van den Heuvel and Sporns, 2011), rich clubs (Harriger et al., 2012; van den Heuvel and Sporns, 2011; Zamora-López et al., 2010), modularity (Chen et al., 2008; Meunier et al., 2009; Schwarz et al., 2008; Sporns and Betzel, 2016), and informational cores (Balduzzi and Tononi, 2008; Kitazono et al., 2018; Kitazono et al., 2020; Tononi, 2004).

Graphical functional connectivity methods are constrained by one-to-one node connections, whereas informational methods can capture many-to-many relationships, which are essential for understanding complex system interactions (Fornito et al., 2016; Rubinov and Sporns, 2010). The concept of the core complex using the informational approach originated from a seminal paper by Tononi (Tononi, 2004), who used a perturbation method to evaluate probability distributions, aiming to quantify actual causation by perturbing a system into all possible states. However, this method requires knowledge of the system’s physical mechanisms, which are often unknown in practice. In this study, we adopted the approach of (Kitazono et al., 2018; Kitazono et al., 2020), which emphasizes that probability distributions can be estimated from empirical data. The core complex framework provides novel insights into network structure and has demonstrated efficiency in systems with a large number of elements and temporal stability (Kitazono et al., 2020). However, previous studies (Kitazono et al., 2018; Kitazono et al., 2020) primarily focused on algorithmic aspects of identifying the core of the system without thoroughly studying its application.

Recent studies have begun to explore the association between core complex structures and cognitive functions, such as consciousness perception and cognition (Taguchi et al., 2024). In that paper, bidirectional network cores have been analyzed using normalized directed transfer entropy (NDTE) to estimate causal interactions between regions. While such approaches emphasize the importance of feedforward and feedback directions in consciousness studies, they rely on causal relationship estimates that may not be well-suited for low temporal resolution data, such as calcium imaging. In contrast, our study employs mutual information to quantify statistical dependence between regions, offering a robust alternative for examining the complex interactions underlying sustained conditioned responses.

In this study, we first employ traditional FC methods to analyze neural interactions during high-response sessions. However, the results were inconsistent across sessions, indicating limitations in capturing the complexity of sustained performance. We then focused on specific regions of interest (ROIs) within the core complex, particularly the PPC, which showed a correlation with enhanced CR sessions. This finding suggests that the PPC may play a role in supporting reward expectations and maintaining consistent high-performance responses across sessions, indicating its potential significance in sustained behavioral engagement.

## 2 Materials and methods

### 2.1 Data and materials

In this study, we utilized the dataset previously reported in Kondo and Matsuzaki (2021), which includes wide-field calcium imaging from 12 region-of-interests (ROIs) of mice during a classical conditioning task with two sound cues assigned to different reward probabilities. In the following, we will briefly describe it, and further details can be found at Kondo and Matsuzaki (2021).

#### 2.1.1 Experimental model and subject details

All animal experiments were approved by the Institutional Animal Care and Use Committee of the University of Tokyo. Mice were housed under standard conditions (food and water ad libitum, 12:12 h light–dark cycle) and included both male and female C57BL/6-derived transgenic lines aged 2–8 months. For wide-field calcium imaging, we used transgenic mice generated by crossing homozygous Emx1-IRES-Cre [B6.129S2- Emx1tm1(cre)Krj/J, JAX stock #005628, Jackson Laboratory (Gorski et al., 2002)] with hemizygous CaMKII-tTA::TITL-R-CaMP1.07 mice. These R-CaMP1.07 mice were originally obtained from Dr. F. Helmchen and are now available as Ai143D from the Jackson Laboratory [though not pre-crossed with CaMKII-tTA mice (Bethge et al., 2017)].

#### 2.1.2 Classical conditioning task

Mice were subjected to a classical conditioning paradigm under controlled body weight conditions (maintained at 80–85% of their initial weight) while receiving food ad libitum. During training, animals were head-fixed and underwent daily sessions of 40–60 min. Two distinct auditory cues (6 kHz or 10 kHz, each lasting 1.5 s) served as conditioned stimuli (CS), followed by a probabilistic delivery of a water reward (unconditioned stimulus, US) after a 0.5 s delay. One cue was associated with a higher reward probability (70%) and the other with a lower probability (30%), with cues presented in randomized order and inter-trial intervals of 8 ± 2 s. Prior to the probabilistic conditioning, mice underwent 2–3 days of pretraining with a guaranteed reward after either cue. Successful learning was defined as a significantly higher licking response (*p* < 0.01, Wilcoxon rank-sum test) for the high-probability cue compared to the low-probability cue in three consecutive sessions. Wide-field calcium imaging was performed throughout the entire training period.

#### 2.1.3 Wide-field calcium imaging

Wide-field calcium imaging was performed in Emx1-Cre::CaMKII-tTA::TITL-R-CaMP1.07 mice following a transcranial preparation. Images were acquired at 30 frames/s (512 × 512 pixels, 2 × 2 binning) using a fluorescent zoom macroscope (Plan-NEOFLUAR Z 2.3×, NA 0.57; Carl Zeiss, Germany) paired with an EM-CCD camera (iXon Ultra 888; Andor Technology, UK). The imaging field encompassed the entire dorsal cortex. An excitation light source (HXP200C; Carl-Zeiss, Germany) with a filter-set (IX3-FGWXL, excitation, 530–550 nm; dichroic, 570 nm; emission, 575 nm LP; Olympus) and barrier filter (FF02-617/73–25,580–655 nm; Semrock, NY) was used to observe R-CaMP1.07 fluorescence. Each session included 54,000 frames (30 min), and no detectable signal fading was observed over multiple days of recording.

#### 2.1.4 Image processing for wide-field calcium imaging data

We analyzed data from six mice that exhibited a statistically significant difference (*p* < 0.05) in anticipatory licking between cue A and cue B trials. All analyses were performed using custom MATLAB routines (R2016a, R2018a; MathWorks, MA). Prior to data processing, each wide-field calcium imaging dataset was rigidly aligned to the dorsal view of the Allen Common Coordinate Framework version 3 (Allen CCF) using 10 anatomical landmarks: the bilateral anterior nexus of the olfactory bulbs, the center and lateral tips of the frontal blood vessels, the lambda suture, and the bilateral posterior and lateral tips of the dorsal cortex. Following alignment, a binary mask derived from the Allen CCF top-view contour was applied to exclude fluorescence signals outside cortical regions.

To reduce computational overhead, only the pixels corresponding to the left hemisphere were retained for subsequent analyses. Noise suppressions were achieved via singular value decomposition (SVD) of the clipped image stack. From the SVD, we obtained spatial components (U), singular values (S), and transposed temporal components ( $V^{T}$ ). The top 12 singular values, which accounted for 99.93% of the original variance of raw data (before extraction of neuronal activities with the ROIs), were selected to reconstruct the image stack with minimal loss of information.

For each pixel in the reconstructed data, changes in fluorescence intensity (ΔF/F) were calculated using the 10th percentile fluorescence value within a ± 15-s window as a baseline. For regional analyses, we defined 12 rectangular regions of interest (ROIs), each measuring 10 × 10 pixels. These ROIs corresponded to previously characterized cortical areas, with stereotaxic coordinates determined according to the Allen CCF. The targeted regions included: dorsomedial frontal cortex (M2, AP +2.8 mm, ML 0.8 mm), anterolateral motor area (ALM, AP +2.5 mm, ML 2.0 mm), primary motor cortex (M1, AP +1.2 mm, ML 1.0 mm), primary sensory forelimb area (S1FL, AP +0.4 mm, ML 2.2 mm), primary sensory hindlimb area (S1HL, AP –0.5 mm, ML 1.5 mm), primary somatosensory cortex mouth area (S1m, AP +1.4 mm, ML 3.0 mm), primary somatosensory cortex nose area (S1n, AP 0 mm, ML 3.4 mm), primary somatosensory cortex barrel area (S1b, AP –1.2 mm, ML 3.0 mm), primary auditory cortex (A1, AP –2.8 mm, ML 4.0 mm), primary visual cortex (V1, AP –4.0 mm, ML 2.2 mm), primary visual cortex (PPC, AP –2.0 mm, ML 2.0 mm), and primary visual cortex (RSC, AP –2.4 mm, ML 0.5 mm). The stereotaxic coordinates indicate the center positions of the ROIs. For each session and mouse, the ROI positions were manually fine-tuned. The fluorescence intensity of each ROI was determined by averaging the included pixels. Neuronal activity extracted from the ROIs was converted into Z-scores and aligned to the onset of each cue-tone.

### 2.2 Functional connectivity

For each condition, we collected the corresponding trials data from each region. The processed region-wise time series of each subject were then trial-averaged to produce 12 nodal time series. Next, we constructed functional connectivity (FC) matrices by computing the Pearson’s correlation coefficient between every possible ROI pair of time series over the entire time course. The resulting values were Fisher z transformed, leading to a 12-by-12 FC matrix per subject. In this paper, we will use FC as an initial approach.

### 2.3 Core complex analysis

To introduce the core complex, we need to define two important concepts: the information loss function and the minimum information partition (MIP). Let a system *V* be composed by *N* elements denoting by 1,2,…,N . We denote $X_{1},X_{2},\ldots ,X_{N}$ as the activities of *N* elements and p as an empirical distribution of the system $p\left(X_{V}\right)=p\left(X_{1},\ldots X_{N}\right)$ . For example, in the case of calcium imaging data, *N* elements are list of ROIs and each $X_{i}$ corresponds to the calcium signal of ROI i .

We consider a subset S⊂V with a given distribution $p\left(X_{S}\right)\text{.}$ A bipartition $\left(S_{L},S_{R}\right)$ of *S* such as $S=S_{L}\cup S_{R}$ and we define a “disconnected” distribution $q\left(X_{S}\right)$ which is produced by removing some connections between elements of $S_{L}$ and $S_{R}$ . The information loss $f\left(X_{S}\right)$ in such a case is quantified in terms of the Kullback–Leibler measure between two distributions $p\left(X_{S}\right)$ and $q\left(X_{S}\right):$


$$f\left(X_{S}\right)=f\left(S_{L},\;S_{R}\right):\;=D_{KL}\left(p\left(X_{S}\right)||q\left(X_{S}\right)\right)$$

There are several information loss functions depending on the types of interaction that could be removed. Here, we only focus on mutual information, which measures the statistical dependence between parts of systems. Within mutual information measurement, we use the conditional independence of a couple of subsystems.


$$q\left(X_{S}\right)=p\left(X_{S_{L}}\right)p\left(X_{S_{R}}\right)$$

In this case, the information loss becomes:


$$\begin{array}{l} f\left(X_{S}\right)=D_{KL}\left(p\left(X_{S}\right)||q\left(X_{S}\right)\right)=\int p\left(X_{S}\right)\;\log\frac{p\left(X_{S}\right)}{q\left(X_{S}\right)}dX_{S}\\ =\int p\left(X_{S}\right)\;\log\frac{p\left(X_{S}\right)}{p\left(X_{S_{L}}\right)p\left(X_{S_{R}}\right)}dX_{S} \end{array}$$

For each bipartition $\left(S_{L},S_{R}\right)$ , there thus exists a corresponding information loss $f\left(S_{L},S_{R}\right)$ . A bipartition $\left(S_{L}^{MIP},S_{R}^{MIP}\right)$ of the subsystem *S* is called Minimum Information Partition (MIP) if for every possible bipartition $\left(S_{L},S_{R}\right)$ within *S*, we have,


$$f\left(S_{L}^{MIP},S_{R}^{MIP}\right)\leq f\left(S_{L},S_{R}\right)$$

In other words, the information loss is minimal at the MIP among all bipartitions of *S*. We denote the information loss value of *S* at its MIP by $f\left(X_{S}^{MIP}\right)\text{.}$

A subsystem S is called a complex if the information loss at its MIP is non-zero and greater than those of all its superset. Mathematically, it means (Def. 1)


$$f\left(X_{S}^{MIP}\right)>0$$

$$f\left(X_{S}^{MIP}\right)>f\left(X_{T}^{MIP}\right)\forall T⊃S\text{.}$$

A subsystem $\bar{S}$ is called a core complex if the information loss at its MIP is non-zero and greater than those of all its superset and greater than or equal to those of all its subsets. Mathematically, it means that (Def. 2)


$$f\left(X_{\bar{S}}^{MIP}\right)>0$$

$$f\left(X_{\bar{S}}^{MIP}\right)>f\left(X_{T}^{MIP}\right)\forall T⊃\bar{S}$$

$$f\left(X_{\bar{S}}^{MIP}\right)\geq f\left(X_{R}^{MIP}\right)\forall R\subset \bar{S}$$

It means that adding or removing an element from a core complex will reduce the information loss. Intuitively, by adding or removing an element from the core complex, it will inevitably create weaker links in the subsystem. Thus, a core complex is the locally most irreducible subsystem, and therefore it can be considered as an informational core of the system.

**Figure 1:**
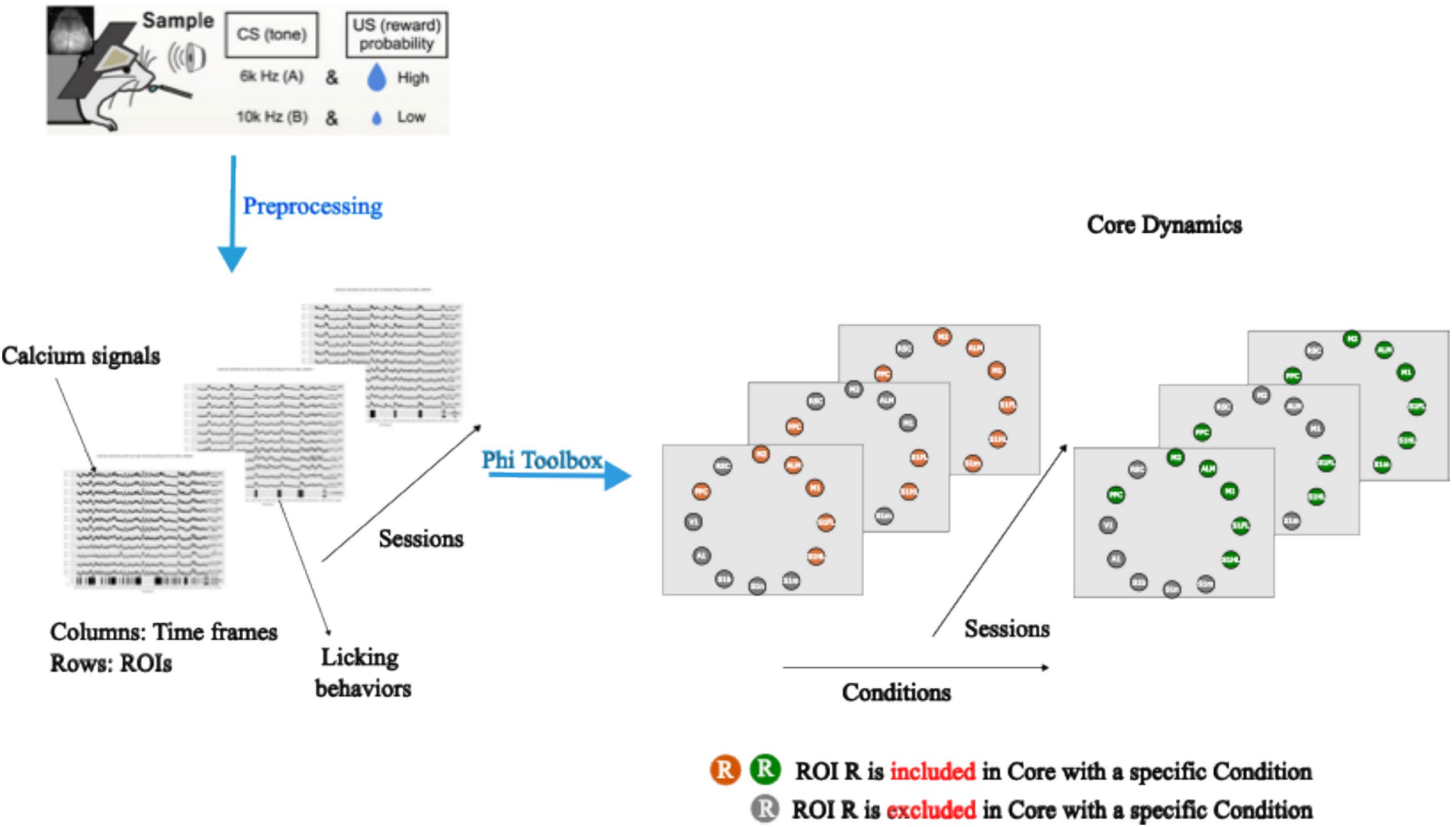
Core complex analysis flows. Step 1: collection, preprocessing of neural activity and behavioral data. Step 2: Core complex dynamics was analyzed using Phi Toolbox, a publicly available MATLAB software package (https://github.com/oizumi-lab/PhiToolbox). For a given condition, data corresponding to that condition were extracted. The core complex (main complex) was determined using the “Complex search” utility in the Phi Toolbox (https://github.com/oizumi-lab/PhiToolbox/blob/master/Complex/Complex_search.m), which computes information loss and identifies the subset of elements forming the core complex. A detailed explanation of this process is provided later in this section. Additionally, a demo of the “Complex search” procedure is available at the following link: https://github.com/oizumi-lab/PhiToolbox/blob/master/demo_Complex_Gauss.m.

**Figure 2:**
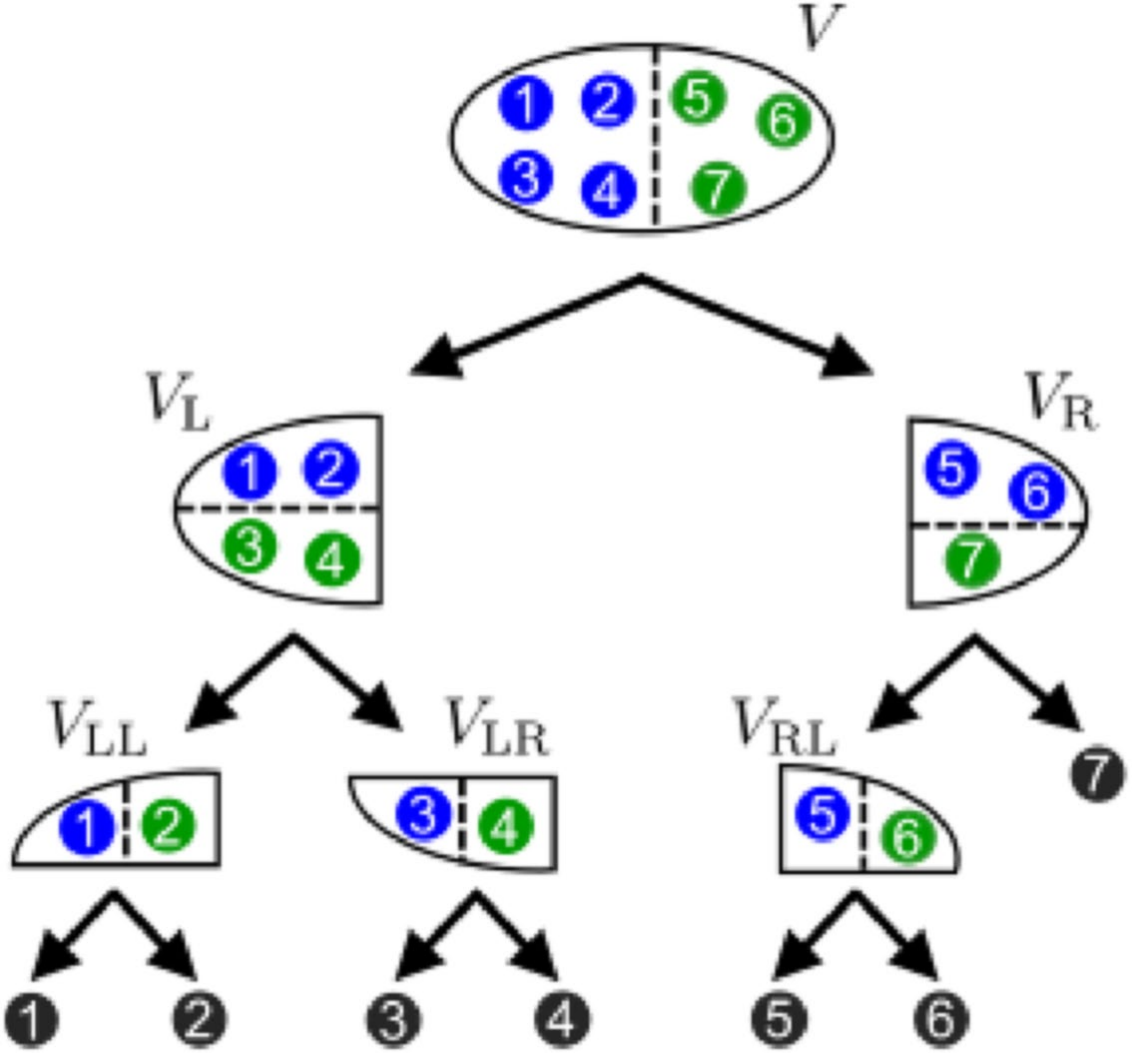
Hierarchical partitioning for complex search (reprinted from Figure 4; Kitazono et al., 2020 according to CC-BY permissions). In this method, a system is iteratively partitioned by its Minimum Information Partitions (MIPs) until it is fully decomposed into individual elements. In this example, the entire system $V=\left\{1,\;\;2,\;\;3,\;\;4,\;\;5,\;\;6,\;\;7\right\}$ is initially divided into two subsystems by its MIP, $V_{L}=\left\{1,2,3,4\right\}$ and $V_{R}=\left\{5,6,7\right\}$ , as indicated by the dashed line. Subsequently, $V_{L}$ is further divided into $V_{LL}$ and $V_{LR}$ , while $V_{R}$ is partitioned into $V_{RL}\;$ and $\left\{7\right\}$ . This process continues until the entire system V is decomposed into its seven individual elements.

In practice, Phi Toolbox,^1^ a publicly available MATLAB software package, was used to identify the core complex of the system (Figure 1). We listed all complex candidates using the HPC algorithm (Hierarchical Partitioning for Complex Search) (Kitazono et al., 2020). This method applies Queyranne’s algorithm to find the MIP (Kitazono et al., 2018) in each partition, which was yet limited to bipartition. As shown in Figure 2 below, the HPC algorithm begins by dividing the entire system based on its MIP. It then iteratively partitions the resulting subsystems using their respective MIPs until the entire system is fully decomposed into individual elements. This hierarchical approach ensures that all possible complex candidates within the system are systematically identified and evaluated.

Moreover, Theorem 8 of Kitazono et al. (2020) shows that the obtained set of subsystems $V=\left\{V,V_{L},V_{R},\ldots \right\}$ (excluding the single elements) contains all the complex candidates. Then by verifying Def. 1, we obtain the list of complexes. The core complex was finally determined as the subsystem whose information loss is locally maximum (Def. 2).

**Figure 3:**
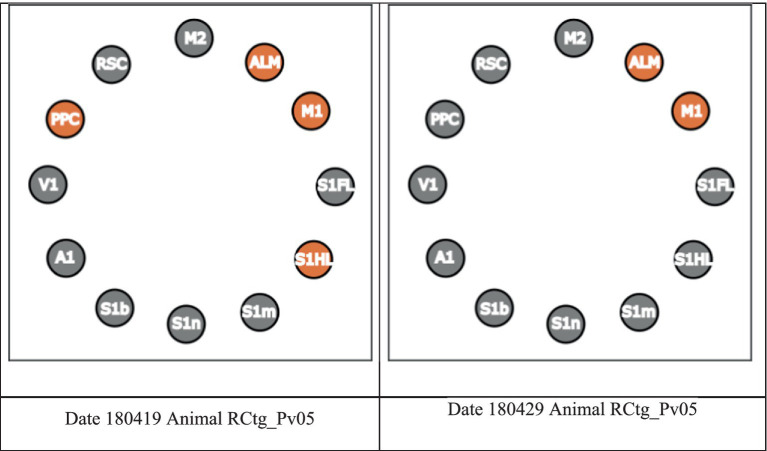
Example of a PPC-included Core session and a PPC-excluded Core session.

In Figure 3, we present the Core using a circular plot. A node in orange indicates that the region is included in the Core. As an example, if we interested in PPC, the Core from session “180419” (left) is referred to as the PPC-included Core, whereas the Core from session “180429” (right) is referred to as the PPC-excluded Core (see Figure 3 below). In short, from now on, we will refer a session whose Core includes (excludes) a region R as a R-included (R-excluded) Core session, respectively.

**Figure 4:**
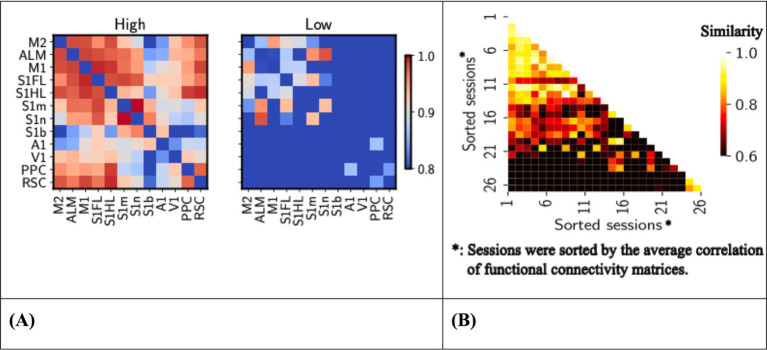
**(A)** Left, functional connectivity between brain regions, session averaged. Heat map showing the Pearson’s correlation coefficients of functional connectivity between various brain regions of high (left) (*n* = 26 sessions) and low (right) response rate sessions (*n* = 8 sessions). High-response rate sessions were sessions whose response rates are above 0.9, whereas low-response rate sessions with response rates were below 0.6. The trial-averaged nodal time series were used to create functional connectivity matrices, with Pearson correlations then Fisher z-transformed. The color scale ranges from 0.8 (low connectivity) to 1.0 (high connectivity). ROIs are labeled on both axes. **(B)** Right, similarity between functional connectivity of high-response sessions. Heat map showing Pearson correlation coefficients between functional connectivity matrices of high response rate sessions (*n* = 26 sessions). Only the upper triangle of functional connectivity matrices was flattened into vectors, and similarity was measured using Pearson’s correlation. The color scale ranges from 0.6 (low similarity) to 1.0 (high similarity). Session numbers are labeled on both axes, sorted by the averaged session values.

### 2.4 Learning indices computation

In this study, we quantified the learning progress of head-fixed mice using response rates. Response rates were measured by the proportion of trials in which mice responded to conditional stimuli (CS) with a higher probability of reward (6 kHz) by performing licking behaviors that we limited to 500 ms around the reward onset. This metric directly reflects the efficacy of learning across the experiment, showcasing the animals’ improvement in recognizing and responding to the CS associated with higher-reward probability trials.


$$ResponseRate=\frac{\;NumberofcueArewardedtrialswithlicks\;}{TotalnumberofcueArewardedtrials}$$

### 2.5 Quantification and statistical analysis

The total sessions for six mice RCtg_Pv05, RCtg_Pv07, RCtg_Pv09, RCtg_Pv10, RCtg_Pv12, RCtg_Pv13 were: 19, 17, 8, 8, 14, 14, respectively. The list of 12 ROIs and their abbreviations were the same as stated in the paper (Kondo and Matsuzaki, 2021). The number of ROI-included Core sessions for cue A and cue B (in parentheses) of six mice is shown in the following table (Table 1):

**Table 1:** The number of ROI-included Core sessions among six animals, sorted by the number of total ROI-included sessions.

Animal ROI	RCtg_Pv05	RCtg_Pv07	RCtg_Pv09	RCtg_Pv10	RCtg_Pv12	RCtg_Pv13	Totals
M1	18 (18)	16 (16)	6 (5)	8 (8)	14 (14)	14 (12)	76 (73)
ALM	16 (16)	15 (15)	8 (6)	4 (4)	7 (10)	9 (10)	59 (61)
M2	9 (9)	10 (12)	2 (1)	8 (8)	14 (14)	14 (12)	57 (56)
S1HL	15 (15)	4 (5)	6 (6)	4 (4)	10 (12)	13 (12)	52 (54)
S1FL	8 (7)	2 (4)	6 (6)	4 (4)	7 (9)	13 (12)	40 (42)
PPC	15 (14)	3 (3)	0 (0)	1 (1)	7 (9)	12 (11)	38 (38)
S1m	3 (2)	4 (6)	8 (6)	1 (1)	5 (7)	2 (2)	23 (24)
S1n	3 (2)	0 (0)	0 (0)	1 (1)	0 (1)	1 (2)	5 (6)
S1b	2 (1)	0 (0)	0 (0)	0 (0)	0 (0)	1 (1)	3 (2)
A1	0 (0)	0 (0)	0 (0)	0 (0)	2 (3)	0 (0)	2 (3)
V1	1 (1)	0 (0)	0 (0)	0 (0)	0 (0)	0 (0)	1 (1)
RSC	0 (0)	0 (0)	0 (0)	0 (0)	0 (0)	0 (0)	0 (0)

The list of 12 ROIs and their abbreviations were the same as stated in the paper (Kondo and Matsuzaki, 2021). The table numbers were the number of sessions that whose Core included a specific ROI for cue A or cue B (in parenthesis) trials.

The Mann Whitney U-Test served as our method for statistical analysis across all distribution pairs, with *p*-values detailed within the legends of the respective figures.

### 2.6 Permutation-based FDR control

To properly evaluate differences of response rates while conducting multiple statistical tests, we applied a permutation-based False Discovery Rate (FDR) control procedure (Westfall et al., 1993). Specifically, we first computed raw *p*-values for each of the comparisons, each designed to assess differences in response rates between two defined groups. To empirically estimate the null distribution, we permuted the sample labels 10,000 times, preserving the original group sizes, and recalculated the *p*-values across all tests for each permutation. Aggregating these permuted p-values produced a distribution reflective of no true differences. From this null distribution, we determined a *p*-value threshold at an FDR level of q = 0.1. The threshold was defined as the largest *p*-value cutoff at which the expected proportion of false positives did not exceed 10%. Any observed *p*-value below this threshold was considered significant under FDR control.

## 3 Results

In this study, we categorized sessions by response rates: high response rate sessions with response rate (for both cues) above 0.9 and low-response rate sessions with response rate (for both cues) below 0.6. These thresholds were selected to ensure adequate session numbers for statistical comparison and to clearly distinguish underlying neural mechanisms. There are 26 high-response rate sessions and 8 low-response rate sessions.

### 3.1 Functional connectivity was not consistent across high response rate sessions

First, we compare the functional connectivity between high- and low-response sessions. High connectivity values were observed between regions such as M2, ALM, and PPC, indicated by hotter shades, reflecting stronger correlations (Figure 4A). These circuits are more apparent in high response rate sessions, suggesting their critical role in supporting superior functions in performing the task. The different patterns of connectivity highlight the importance of these areas of the brain in achieving a high response rate, providing insight into the neural substrates that underlie potentially motivated behavior.

Next, we measured the similarity between functional connectivity matrices within the high response rate sessions. The generally low correlation values, predominantly around 0.6 in Figure 4B, indicate significant variability in functional connectivity patterns between these sessions. This suggests that despite showing high response rates, the underlying neural connectivity does not consistently conform to a uniform pattern, highlighting the complexity and variability in the neural mechanisms that support highly motivated behavior or actively respond in the task. Therefore, in the following sections, we will investigate a core sub-network that might be the key to maintaining a high response rate to the CS.

### 3.2 Number of appearances of the region of interest within the core complex

**Figure 5:**
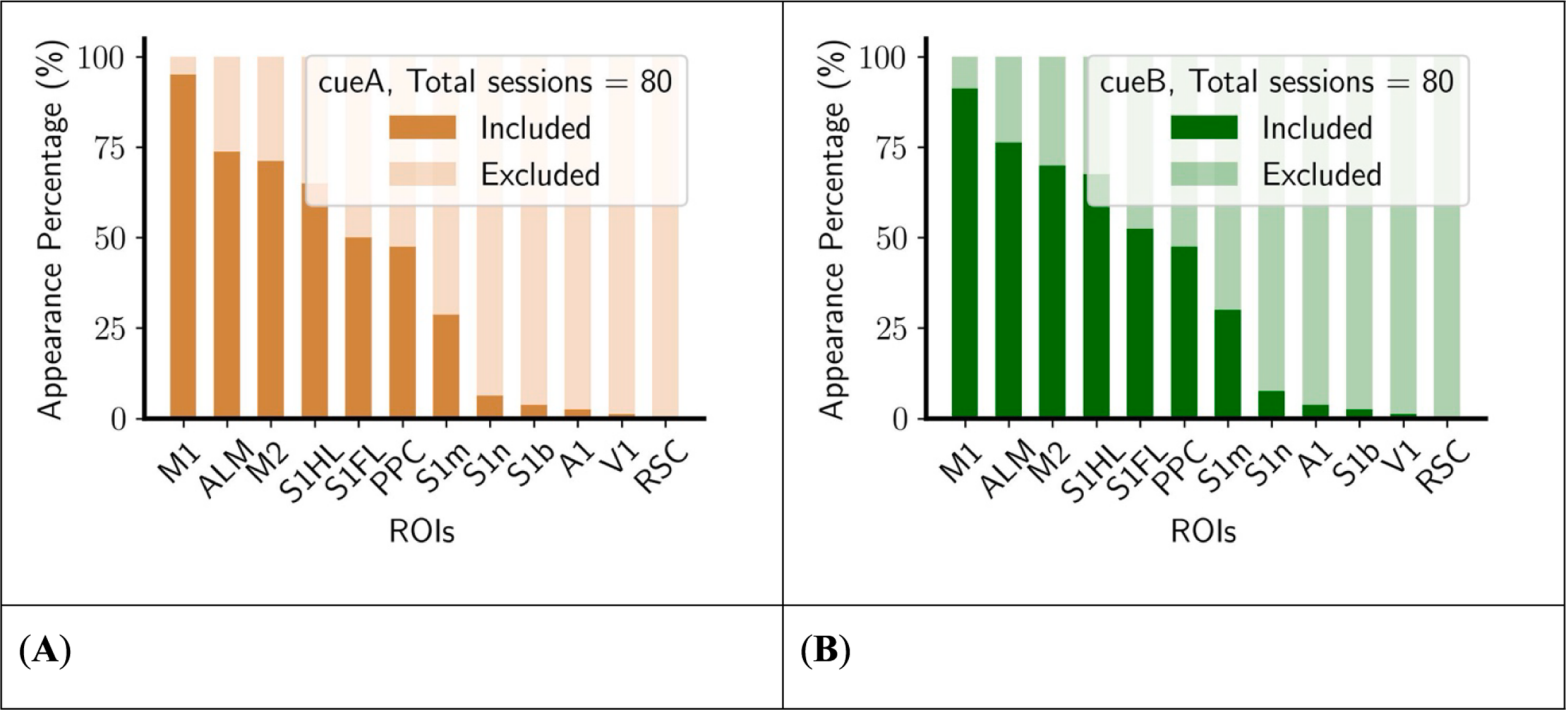
M1 is mostly included in the core complex, whereas some ROIs (S1n, S1b, A1, V1, RSC) are mostly excluded in the core complex. **(A)** Left, cueA trials. **(B)** Right, cueB trials. The number of appearances was counted across all animals during training sessions (see Table 1 for details), sorted in descending order of the total appearance number.

To evaluate the functional significance of specific regions within core complex, we counted the present value of each ROI in core complexes (Figure 5). Certain regions exhibited biases associated with their presence in the dynamic of the core complex. For example, the retrosplenial cortex (RSC) is consistently not involved in the core complex, while the primary motor cortex (M1) is predominantly included. Furthermore, there are only a limited number of sessions whose Core includes the primary somatosensory cortex nose area (S1n), the primary somatosensory cortex barrel area (S1b), the primary auditory cortex (A1), or the primary visual cortex (V1), rendering the data set insufficient for reliable statistical comparisons regarding its inclusion or exclusion in the core complex. As a result, these regions were not examined in the following analyses.

### 3.3 Inclusion of PPC in the core complex is associated with high response rates

In this section, we sought to determine whether the presence of certain regions in the core complex might increase response rates. We assessed the learning progress of mice by evaluating their response rates to trials with a high reward probability (cue A trials) in the initial stage of experiment (first half), calculated as the licking trials over the total number of rewarded cue A trials (See Methods section). We focused exclusively on the initial stage of learning because we aim to investigate the influence of certain brain regions on learning before it becomes fully established (Supplementary Figure S1).

**Figure 6:**
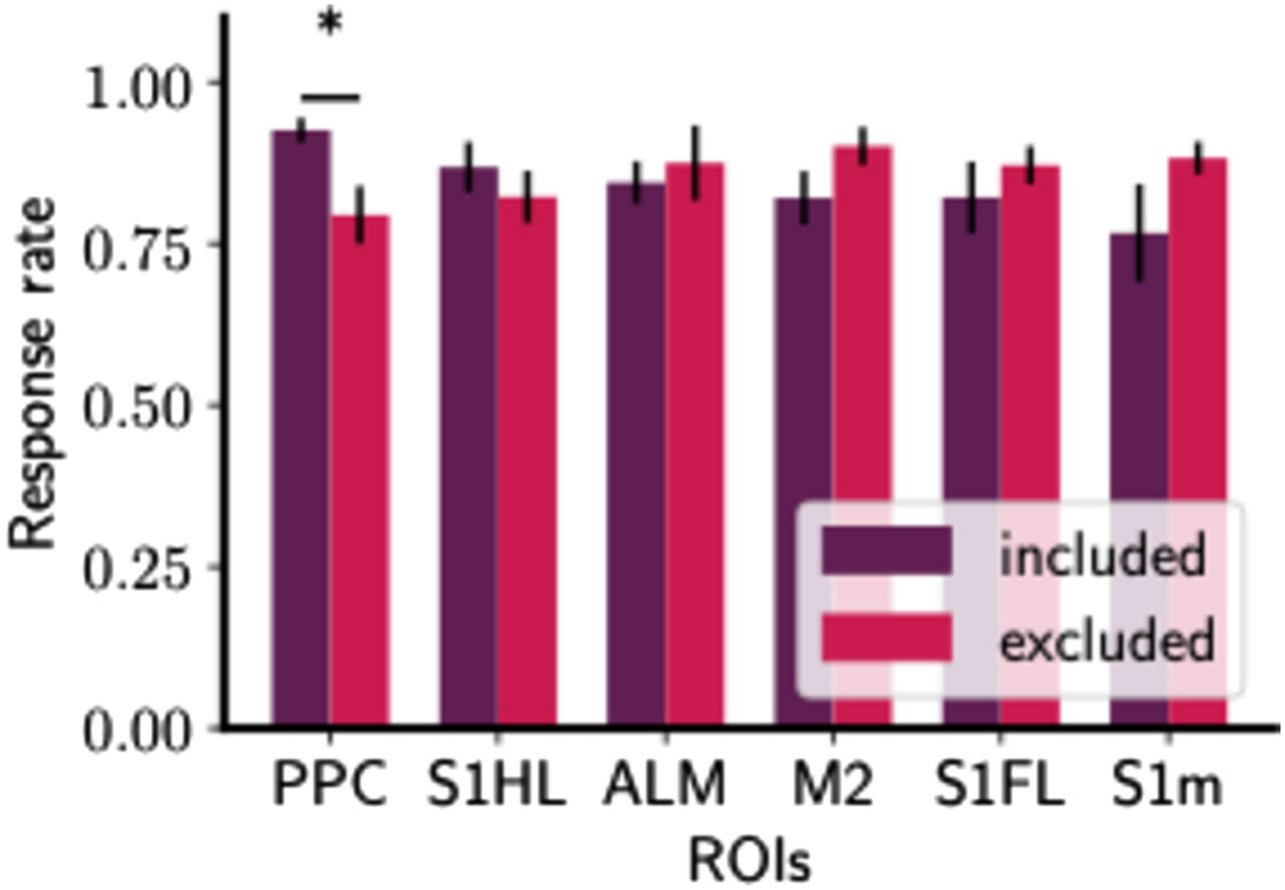
The core complex with PPC shows higher response rates in the initial learning phase. Bar plots of response rates with included and excluded of a certain ROI. Response rates were measured by the proportion of rewarded cue A trials in which mice correctly licking around reward timing. For each session, the Core complex was determined using data in the cue A condition. Then, for each ROI (R), the Core complexes were categorized into two groups: Included (Core complex includes R) and Excluded (Core complex does not include R). Bar plots as Mean ± S.E.M. The number of ROI-included Core sessions was provided in Supplementary Table S1. The differences in response rate between each pair of groups were analyzed using a two-sided Mann–Whitney *U*-test with FDR control (see Section 2.6). Significant differences under FDR control are indicated by asterisks. See Supplementary Table S3 for further detail of statistical tests.

As shown in Figure 6, the inclusion of PPC in the core complex sessions correlates with a significant increase in the response rate (U-statistic = 245.5, *p* = 0.0182), reflecting an elevated likelihood of responses. We also checked this result against our permutation-based FDR control at q = 0.1, confirming that it remained significant and not solely attributable to multiple testing. On the contrary, sessions that omit the involvement of the secondary motor cortex (M2) or the anterolateral motor area (ALM) are associated with elevated response sessions. This suggests that while the inclusion of PPC may play a role for learning enhancement, the absence of M2 or ALM in the core complex does not hinder and may even benefit, task performance in the initial learning stage. However, this increase in response rate associated with the PPC-included Core was not observed during the later stages of training (Supplementary Figure S2), suggesting its specific role in the early phase of learning.

## 4 Discussion

Recent research on auditory decision-making has highlighted the pivotal role of the auditory cortex (A1) in encoding both cue and target information (Napoli et al., 2021). While A1 demonstrates robust and temporally precise neuronal activity, its role is relatively transient (Runyan et al., 2017). A1 is foundational in encoding sensory details, which are then integrated by higher-order regions like the dorsolateral prefrontal cortex (dlPFC) and basolateral amygdala (BLA), which are more directly involved in decision-making at the moment of choice (Banno et al., 2020; Napoli et al., 2021). However, this initial sensory processing is insufficient to account for sustained behavioral engagement across repeated sessions. The posterior parietal cortex (PPC), in contrast, plays a more sustained role, encoding choice information for longer durations and exhibiting stronger neuronal coupling than A1 (Banno et al., 2020; Runyan et al., 2017). This suggests that while A1 is critical for early auditory processing, the PPC is integral for maintaining and integrating information over time to guide decision-making.

In the context of classical conditioning, where a conditioned stimulus (CS) is paired with an unconditioned stimulus (US), the mechanisms that sustain high conditioned response (CR) rates across repeated sessions remain poorly understood. Previous studies have identified several regions involved in encoding reward-predicting cues and reward processing, yet less is known about how cortical regions maintain consistent performance over time (Bloem et al., 2017; Kondo and Matsuzaki, 2021). In particular, the role of cortical regions in linking primary sensory input with motor output and how reward expectation is represented on a session-by-session basis remain unclear.

In this study, we adopted an innovative network analysis method, the core complex framework (Kitazono et al., 2018; Kitazono et al., 2020), which focuses on highly interconnected regions that support sustained cognitive functions. Our analysis suggests that the PPC might be involved in maintaining high CR across sessions. However, in this study, the limited number of subjects and session data led to PPC significance in Figure 6 only emerging at an FDR of 10%. Thus, further analysis is needed to clarify the PPC’s role in supporting reward expectations and maintaining consistent behavioral responses. In addition, it is important to note that the neural mechanisms examined in this study were limited to the dorsal cortex, not including subcortical regions such as the striatum.

The core complex was first introduced in Tononi (2004) seminal paper on consciousness studies. It uses mutual information to measure statistical dependence between brain areas, capturing both pairwise and many-to-many relationships, as well as linear and nonlinear dependencies, making it suitable for evaluating complex cognitive functions (Fornito et al., 2016; Li, 2022). Although Tononi’s original approach employed perturbation methods requiring knowledge of the system’s physical mechanisms to estimate probability distributions, our study uses the core complex approach from (Kitazono et al., 2018; Kitazono et al., 2020), which estimates probability distributions directly from raw data. Furthermore, the core complex has demonstrated temporal stability in EEG data (Kitazono et al., 2020). For slow temporal resolution data, such as calcium imaging, where Granger causality is less effective, core complex analysis offers an alternative for capturing essential network structures. However, one of the most apparent flaws of this approach is its inability to provide dynamic structures at full scale. In addition, although network analysis using mutual information provides complete interactions between brain regions, interpreting the results from statistical and neuroscientific perspectives remains challenging (Fornito et al., 2016; Li, 2022). Another technical limitation of the core complex analysis is its reliance on the assumption that the data roughly obey a Gaussian distribution, which is often not met in real-world data. This issue can be addressed by employing a discrete symbol transformation approach (Bandt and Pompe, 2002; King et al., 2013), which converts continuous signals into discrete signals, allowing analysis using PyPhi (Mayner et al., 2018), a toolbox designed for the integrated information theory framework (IIT 4.0).

Future research should further explore the use of PyPhi to study the involvement of consciousness in general framework such as decision-making. Another important direction would be to conduct experiments that inactivate PPC or disrupt its connections to other brain regions to thoroughly examine changes in core complex dynamics and their impact on task performance. These studies will deepen our understanding of the PPC’s role in neural computations underlying decision-making.

## Data Availability

The data analyzed in this study is subject to the following licenses/restrictions: the datasets and code that support the findings of this study are available from TCP and MM, upon reasonable request. Requests to access these datasets should be directed to mzakim@m.u-tokyo.ac.jp.

## Footnotes

^1^ https://github.com/oizumi-lab/PhiToolbox

## Supplementary material

Supplementary Figure S1 Response rates (cueA) of all animals.

Supplementary Figure S2 Response rates in the second half of the training. Format is the same as Figure 6.

Supplementary Table 1 The number of ROI-included/excluded Core sessions in the first half of the training.

Supplementary Table 2 The number of ROI-included/excluded Core sessions in the second half of the training.

Supplementary Table 3 Mann Whitney statistical test values in Figure 6.
