# Supplementary figures and images for: Integrated information theory reveals the potential role of the posterior parietal cortex in sustaining conditioning responses in classical conditioning tasks
Source: Front Neurosci. 2025 Jan 29;19:1512724. doi: 10.3389/fnins.2025.1512724 (PMC11814451; doi:10.3389/fnins.2025.1512724)

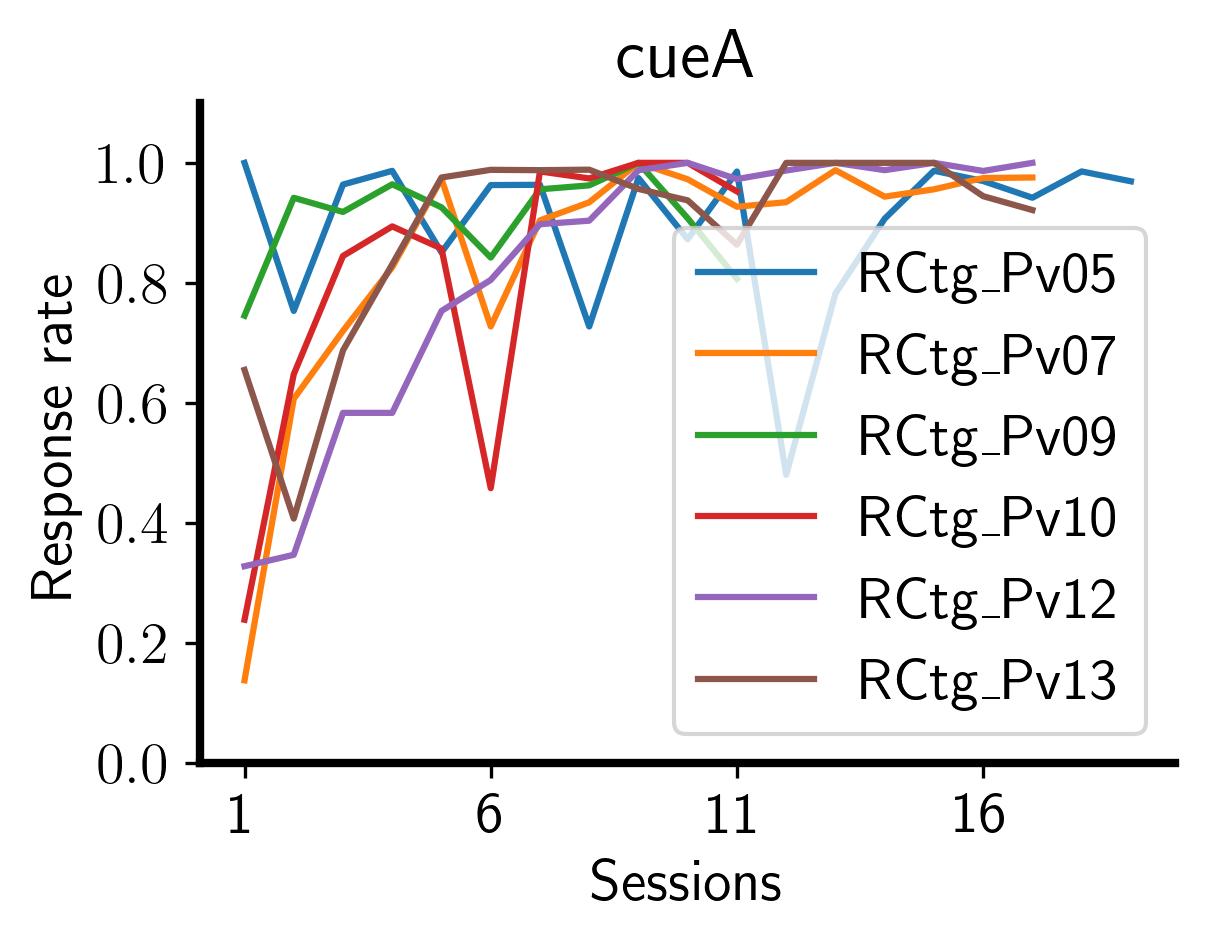

Supplement: Supplementary Figure S1 — Response rates (cueA) of all animals. [file Image_1.JPEG]

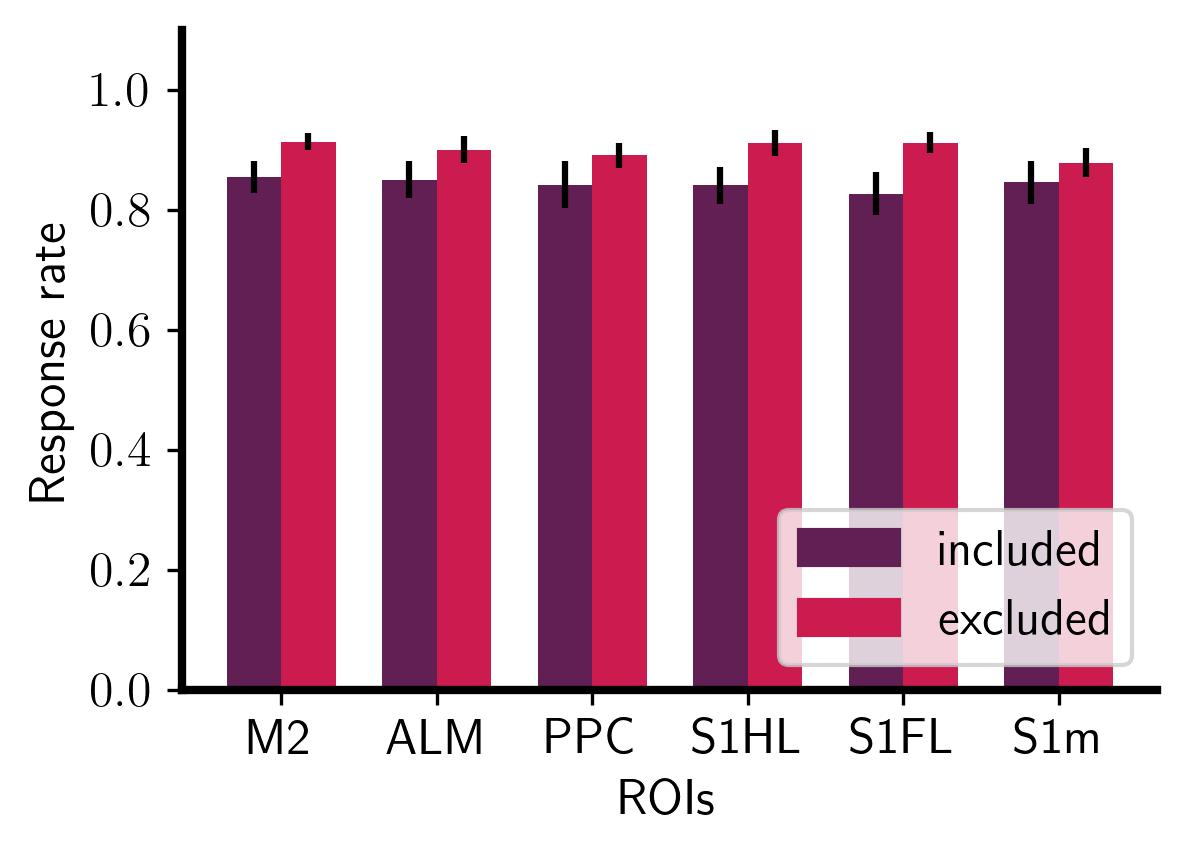

Supplement: Supplementary Figure S2 — Response rates in the second half of the training. Format is the same as Figure 6. [file Image_2.JPEG]
